# Metformin Inhibits Lipoteichoic Acid–Induced Oxidative Stress and Inflammation Through AMPK/NRF2/NF-κB Signaling Pathway in Bovine Mammary Epithelial Cells

**DOI:** 10.3389/fvets.2021.661380

**Published:** 2021-06-28

**Authors:** Abdelaziz Adam Idriss Arbab, Xubin Lu, Ismail Mohamed Abdalla, Amer Adam Idris, Zhi Chen, Mingxun Li, Yongjiang Mao, Tianle Xu, Zhangping Yang

**Affiliations:** ^1^College of Animal Science and Technology, Yangzhou University, Yangzhou, China; ^2^Darfur College, Biomedical Research Institute, Niyla, Sudan; ^3^Joint International Research Laboratory of Agriculture and Agri-Product Safety of Ministry of Education of China, Yangzhou University, Yangzhou, China

**Keywords:** metformin, AMPK signaling, antioxidant, anti-inflammation, bovine mammary epithelium cells

## Abstract

The objective of this research was to explore the effect of metformin on the lipoteichoic acid (LTA)–induced mastitis model using isolated primary bovine mammary epithelial cells (PBMECs). The PBMECs were exposed to either 3 mM metformin for 12 h as a metformin group (MET) or 100 μg/mL LTA for 6 h as LTA group (LTA). Cells pretreated with 3 mM metformin for 12 h followed by washing and 100 μg/mL LTA exposure for 6 h served as the MET + LTA group. Phosphate-buffered saline was added to cells as the control group. PBMECs pretreated with different metformin doses were analyzed by a flow cytometry (annexin V–fluorescein isothiocyanate assay) to detect the cell apoptotic rate. We performed quantitative reverse transcriptase–polymerase chain reaction and Western blot analysis to evaluate the inflammatory and oxidative responses to metformin and LTA by measuring cellular cytotoxicity, mRNA expression, and protein expression. Immunofluorescence was used to evaluate nuclear localization. The results showed that the gene expression of *COX2, IL-1*β, and *IL-6* significantly increased in the cells challenged with LTA doses compared to control cells. In inflammatory PBMECs, metformin attenuated LTA-induced expression of inflammatory genes nuclear factor κB (NF-κB) p65, tumor necrosis factor α, cyclooxygenase 2, and interleukin 1β, as well as the nuclear localization and phosphorylation of NF-κBp65 protein, but increased the transcription of nuclear factor erythroid 2–related factor 2 (Nrf2) and Nrf2-targeted antioxidative genes heme oxygenase-1 (HO-1) and Gpx1, as well as the nuclear localization of HO-1 protein. Importantly, metformin-induced activation of Nrf2 is AMP-activated protein kinase (AMPK)–dependent; as metformin-pretreated PBMECs activated AMPK signaling via the upregulation of phosphorylated AMPK levels, cell pretreatment with metformin also reversed the translocation of Nrf2 that was LTA inhibited. This convergence between AMPK and Nrf2 pathways is essential for the anti-inflammatory effect of metformin in LTA-stimulated PBMECs. Altogether, our results indicate that metformin exerts anti-inflammation and oxidative stress through regulation of AMPK/Nrf2/NF-κB signaling pathway, which highlights the role of AMPK as a potential therapeutic strategy for treatment of bovine mastitis.

## Introduction

Mastitis is a frequent and costly bovine mammary disease in the milk production industry (1), which seriously affects animal health and production ability and causes a huge economic loss to the dairy industry (2). *Staphylococcus aureus* is a frequently isolated Gram-positive bacteria that induce mastitis (3, 4). Approximately one-third of clinical and subclinical mastitis cases are caused by *S. aureus* infection in dairy cattle, characterized by less severe inflammation and is sometimes asymptomatic (5). Lipoteichoic acid (LTA), a bacterial endotoxin embedded in the cytoderm of *S. aureus*, is a critical factor known to activate inflammatory responses (6) and affect lactation in the mammary gland of cows (7, 8). Toll-like receptors (TLRs), particularly TLR2, are involved in LTA detection derived from Gram-positive bacteria (9, 10), and TLR2 activation leads to nuclear factor κB (NF-κB) activation, which regulates the expression of proinflammatory cytokines, such as *IL1B, TNFA*, and *IL6* (11).

AMP-activated protein kinase (AMPK), a sensor of intracellular energy status, is an attractive target for suppressing inflammation. Indeed, evidence shows that AMPK activation can decrease oxidative stress and inhibit inflammation (12). However, mechanistic connections between AMPK and inflammation have limited links with the NF-κB pathway (13). NF-κB and mitogen-activated protein kinase signaling pathways regulate cytokines and chemokine expression, which are essential immune mediators during inflammation (14). On sensing redox system imbalance, AMPK exerts a beneficial effect in the prevention of reactive oxygen species (ROS) accumulation to alleviate oxidative stress (15, 16). Notably, the AMPK pathway shares distinct crosstalk with the antioxidant response, specifically, nuclear factor erythroid 2–related factor 2 (Nrf2). Recently, the direct phosphorylating effect of AMPK on Nrf2 has been identified (17). Many studies have supported the notion that the Nrf2 antioxidant pathway is downstream to AMPK (18, 19). Whether AMPK plays a positive action in inhibiting oxidative stress and inflammation in bovine mammary epithelial cells (BMECs) remains unknown.

To date, treatment and prevention of bovine mastitis relied on antibiotics. However, the growing concerns about antibiotic therapies being linked with the emergence of drug-resistant bacteria, have found the industry to look to alternative safe and available antibacterial treatment. Metformin (Met), a derivative of biguanide, was initially developed from natural compounds found in the plant *Galega*. It is officially known as French lilac or goat's rue and is one of the most classic and standard first-line therapies commonly used to treat type 2 diabetes for nearly 60 years (20). Met exerts its effect through targeting multiple pathways such as activating AMPK and inhibiting the mTOR, HER2, and NF-κB pathways (21). Studies indicate the dual role of AMPK activation using its inducers such as Met on the tumor necrosis factor α (TNF-α) levels in different tissues of rats (22). In mice, experiments indicated that induction of AMPK by its inducers leads to the inhibition of *COX2, TNF*α, *IL-6*, and *iNOS* through suppressing NF-κB nuclear translocation in neurons (23). Studies in the rat reported that Met activates Nrf2 in an AMPK-dependent manner and exerts antioxidant and anti-inflammatory effects under global cerebral ischemia; moreover, pretreatment with Met enhanced the level of glutathione and catalase activities compared with those in the ischemic group (24). Activation of AMPK by Met stabilized Nrf2 levels, and this result leads to the protective role of Met in oxidative stress. Therefore, pretreatment with Met increased Nrf2 expression sufficiently to induce antioxidant response element (ARE) genes, which subsequently activate antioxidant related factors such as heme oxygenase-1 (HO-1), glutathione, and catalase (25). In this study, we aimed to investigate the potential effect of Met on the LTA-induced mastitis model using isolated BMECs, with respect to the activation of the AMPK signaling pathway and inhibition of inflammatory responses and oxidative stress through the suppression of NF-κB and Nrf2 signaling.

## Materials and Methods

### Materials

Met was purchased from Sigma (D150959; Sigma–Aldrich, St. Louis, MO, USA) with a purity of >97%. LTA (derived from *S. aureus*) used in these experiments was purchased from Sigma (L2515; Sigma–Aldrich).

### Ethics Statement

All experimental procedures were approved by the Animal Experiment Committee of Yangzhou University (YZUDWLL-202003-209), following the Regulations for the Administration of Affairs Concerning Experimental Animals (The State Science and Technology Commission of China, 1988) published by the Ministry of Science and Technology, China, in 2004. All of the experimental protocols were performed in accordance with the approved guidelines and regulations.

### Primary BMEC Isolation and Culture

Mammary gland biopsy was selected from three peak lactation dairy cows (26). After phosphate-buffered saline (PBS) washing, fat tissue and connective tissue were removed. BMECs were separated by the tissue block method followed by purification via differential digestion and cryopreservation after subculturing. Cells were incubated in Dulbecco modified eagle medium (DMEM)/F12 supplemented with 10% (vol/vol) fetal bovine serum including 5 μg/mL bovine insulin and 10 KU/L cyan/streptomycin. The resuscitated mammary epithelial cells (MECs) were cultured in an incubator at 37°C, 5% CO_2_, and suitable humidity. The medium was changed every 48 h. The BMECs were digested with 0.25% trypsin for passaging, and the growth of cells was observed using an inverted microscope (26, 27).

BMECs are primary cells isolated from the mammary glands of dairy cows. The primary cells can genuinely reflect the situation in the cow. To our knowledge, research of lactation function in MECs is mainly on milk protein (the main component is casein) gene expression. β-Casein is a marker protein representing the lactating function of MEC (28). Our research group who determined both mRNA level and protein level of β-casein in BMECs indicated that BMEC possesses secretory capacity. Therefore, confirmation of BMECs maintained lactating functions and can study gene expression of milk protein and milk secretion mechanism in the mammary gland.

The culture of primary bovine mammary epithelial cells (PBMECs) was performed as described previously (26, 29). Briefly, mammary tissue obtained from three lactating dairy cows without incidence of clinical disease was used for cell isolation and purification. The BMECs cultured in basal medium contained DMEM/F12 (catalog number: 11320082, American Thermo Fisher, Waltham, MA, USA) and 10% fetal bovine serum, and various cytokines (e.g., 5 μg/mL bovine insulin, 10 kU/L cyan/streptomycin) (catalog no. 7120-30; Invitrogen, Carlsbad, CA, USA). The resuscitated BMECs were cultured at 37°C, 5% CO_2_, and proper humidity. When the cell confluence reached 80%, after infection of small RNA chemical synthesis reagents, cells were collected 48 h later for the following analyses. Three replicates for each treatment were used. All experiments were performed with cells at the four to six passages. Cells (2 × 10^5^) were seeded in six-well plates with overnight incubation in complete medium (90% RPMI 1640, 8119417; Gibco, CA, USA), 10% fetal bovine serum, and antibiotics (penicillin 100 IU/mL; streptomycin 100 μg/mL). All medium supplements were from Gibco (Thermo Fisher Scientific, CA, USA). The cells were maintained at 37°C and 5% CO_2_ in a humidified incubator until reaching confluence.

### Experimental Design

**Figure 1:**
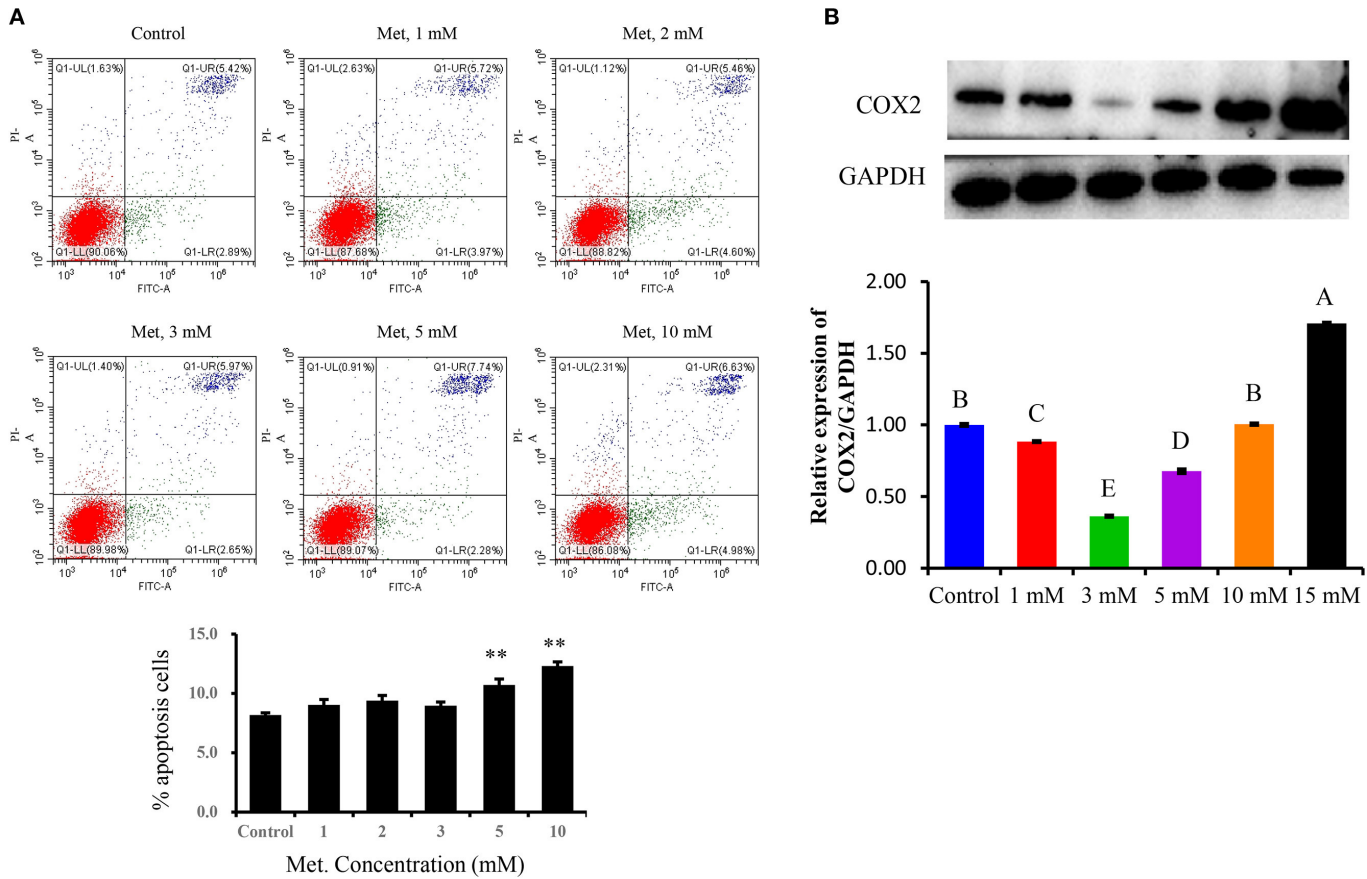
Determination of the optimal metformin concentration in the study. Metformin was used to investigate its effect on growth and apoptosis in PBMECs. PBMECs were pretreated with the indicated concentrations of metformin (1, 2, 3, 5, and 10 mM) and incubated for 12 h; apoptotic cells were measured using flow cytometry, a statistical graph of the apoptotic death rates is shown in **(A)**. Western blot analysis was used to determine the optimum concentration of metformin among (1, 3, 5, 10, and 15 mM) metformin doses **(B)**. Values represent means ± SEM, different uppercase letters (A–E) on the top of bars indicate significant differences (*n* = 3, *p* < 0.05). ^**^indicates *p* < 0.01 vs the Control group.

**Figure 2:**
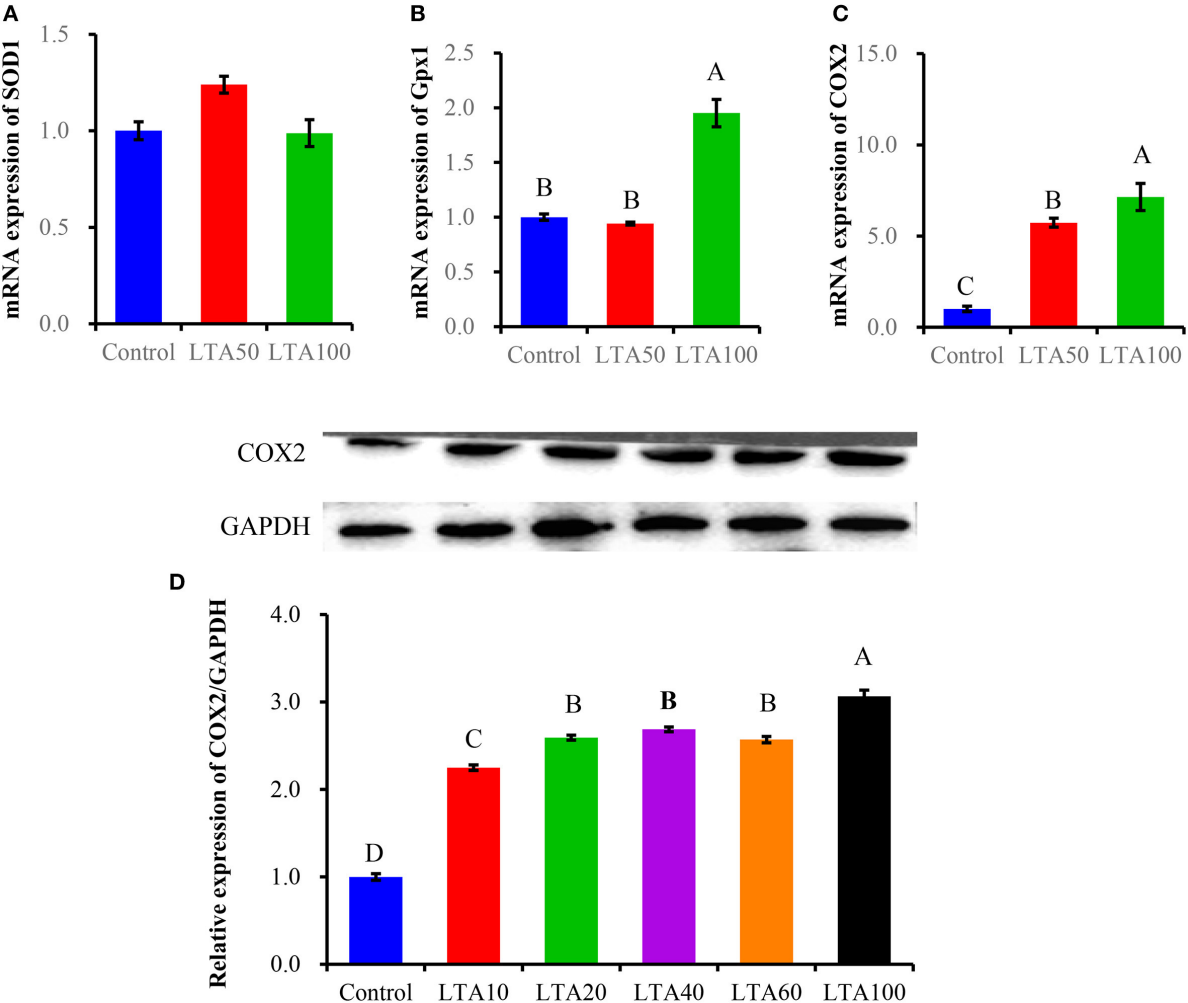
Dose-dependent effect of LTA in PBMECs. Effect of lipoteichoic acid on SOD1 and Gpx1 antioxidant enzyme and COX2 expressions measured in PBMECs to determine the suitable LTA dose by qRT-PCR **(A–C)**. For optimum LTA dose confirmation, the expression of COX2 protein was measured in PBMECs stimulated with LTA (100 μg/mL) **(D)**. Glyceraldehyde-3 phosphate dehydrogenase (GAPDH) is used as a control. Graphs show the densitometric analysis (protein/GAPDH) from three independent experiments. Values represent means ± SEM. LTA, lipoteichoic acid. Different uppercase letters (A–D) on the top of bars indicate significant differences (*n* = 3, *p* < 0.05) among the groups.

The inflammation model was tested and optimized concerning LTA and Met concentrations. Dose dependence for Met to choose a suitable treatment for the following experiments was as follows: PBMECs pretreated with graded concentrations of Met (1, 2, 3, 5, and 10 mM) for 12 h were used to investigate the apoptosis rate by flow cytometry analysis (Figure 1A). Also, Western blot analysis was used to determine the optimum concentration of Met among Met doses of 0, 1, 3, 5, 10, and 15 mM (Figure 1B). Quantitative reverse transcriptase–polymerase chain reaction (qRT-PCR) and Western blot analysis were used to measure LTA dose-dependence (Figure 2): Western blot analysis for PBMECs induced by LTA with various concentrations (0, 10, 20, 30, 40, and 100 μg/mL) for 6 h. For qRT-PCR, PBMECs were induced by LTA doses (0, 50, and 100 μg/mL) for 6 h, as shown in Figures 2A–C.

PBMECs were challenged with LTA to stimulate the *in vitro* setting that existed in mastitis. A total of 5 μg of LTA was dissolved in 5 mL of RPMI 1640 (CCM) to serve as a stock solution (1 μg/mL). Four treatments were set as follows: a control group (Control) supplied with PBS only; LTA treatment refers to the cells administered with LTA at a concentration of 100 μg/mL for 6 h; cells pretreated with Met were subsequently primed with or without LTA as the MET + LTA group or MET group. For Met treatments, the PBMECs were treated with Met at a dose of 3 mM according to the dose-dependent assay for 12 h.

### Flow Cytometry Analysis

The apoptotic status of PBMECs was evaluated by measuring the exposure of phosphatidylserine on the cell membranes using annexin V–fluorescein isothiocyanate (FITC) and propidium iodide (PI) staining [50]. The BD Pharmingen annexin V–FITC Apoptosis Detection Kit I (BD Biosciences, Franklin Lakes, NJ, USA) was used for the apoptosis assay. PBMECs (2 × 10^4^ cells/well) were plated in a 12-well plate, and after 24-h incubation, the cells were treated with graded concentrations of Met (0, 1, 2, 3, 5, and 10 mM) for 12 h and harvested. The cell pellets were washed twice after centrifugation with cold PBS (137 mM NaCl, 2.7 mM KCl, 10 mM Na_2_HPO_4_, pH 7.4) and suspended in 100 μL of 1 × binding buffer (10 mM HEPES/NaOH, 140 mM NaCl, 2.5 mM CaCl_2_, pH 7.4). The cells were incubated with 5 μL annexin V–FITC and 10 μL PI at room temperature for 15 min in the dark. After the incubation, 400 μL of 1 × binding buffer was added to each tube. The cells were analyzed immediately by Epics XL-MCL Flow Cytometry (Beckman–Coulter, Cassina de Pecchi, Italy).

### RNA Isolation, cDNA, and Gene Expression Using Quantitative Real-Time PCR Analysis

After removing the cell culture, the cells were washed with PBS twice, and the TRIzol reagent (catalog no. 9108; Takara, Dalian, China) was added to isolate the total RNA in cells, and a NanoDropND-1000 spectrophotometer (Thermo Fisher Scientific Inc., Waltham, MA, USA) was used to value the concentration and quality of the isolated RNA. One microgram of total RNA was added in the reaction system to reverse transcribe into cDNA by a Prime Script RT Master Mix Kit (catalog no. RR036A; Takara), as described in the manufacturer's instructions. Related primers were designed with Premier 6.0 software (Premier Biosoft International, Palo Alto, CA, USA), and the primer sequences were provided in Table 1. The efficiency of primers was evaluated before use. Each cDNA sample was amplified through qRT-PCR using the SYBR Premix Ex Taq Kit (catalog no. DRR420A; Takara) on an ABI 7300 Fast Real-time PCR system (Applied Biosystems, Foster City, CA, USA). Glyceraldehyde phosphate dehydrogenase (*GAPDH*), *RPS9*, and *UXT* were used as an internal control of the mRNA expression in our experiments. Fold changes of the related mRNAs were quantified by the 2^−ΔΔCt^ method (30).

**Table 1:** Sequence of primers used in this study.

**Genes**	**Sequence (5^**′**^-3^**′**^)**	**References**
Gpx1	F: CCCCTGCAACCAGTTTGG, R: GAGCATAAAGTTGGGCTCGAA	
SOD1	F: GGCTGTACCAGTGCAGGTCC, R: GCTGTCACATTGCCCAGGT	
COX2	F: CGTTTTCTCGTGAAGCCCTATG, R: CTCCATGGCATCTATGTCTCCAT	
GAPDH	F: GGGTCATCATCTCTG CACCT, R: GGTCATAAGTCCCTCCACGA	(29)

*The reverse transcription–generated cDNA encoding Gpx1 (glutathione peroxidase 1), SOD1 (superoxide dismutase-1), COX2 (cyclooxygenase 2), GAPDH (glyceraldehyde-3 phosphate dehydrogenase) was amplified by RT-PCR using the above selective primers. F, forward primer (5′-3′); R, reverse primer (3′-5′)*.

### Western Blot Analysis

Western blot was performed using the protocols described previously (26). Briefly, PBMECs were lysed in RIPA lysis buffer (Beyotime Biotechnology, Shanghai, China) and subjected to 10% sodium dodecyl sulfate–polyacrylamide gel electrophoresis gels and then were transferred onto nitrocellulose (NC) membranes (Bio Trace, Pall Corp, Port Washington, NY, USA). After blocking with 7% skim milk or 5% bovine serum albumin (BSA) [used for phosphorylated protein) in Tris-buffered saline with Tween (TBST) for 2 h at room temperature, the NC membranes were incubated overnight at 4°C with the primary antibodies for P-AMPK, AMPK, cyclooxygenase 2 (COX-2), P65, P-NRF2, HO-1, Gpx1, and GAPDH]. Rabbit antibodies were purchased from Beyotime (#AA393-1, #AF1627, #AF1924, #AF1234, #AF1609, #AF1333, #AF0162, and AF1186). Interleukin 1β (IL-1β) was purchased from Bioss ANTIBODIES (AL08133511/bs-6319R). P-P65 (#3033) and NRF2 (#1271T) were purchased from Cell Signaling Technology (Danvers, MA, USA). After washing with TBST, six times 5 min each the next day, the blots were incubated at room temperature for 2 h with corresponding goat anti-rabbit secondary antibody, purchased from Abcam (ab6712), and were diluted 1:5,000. After washing with TBST six times 5 min each and chemiluminescence, bands were detected and analyzed with Bio-Rad Gel Doc 2000 system analysis software (Bio-Rad, Hercules, CA, USA). GAPDH was used as a reference in our present study. The protein expression was quantitatively analyzed using ImageJ software.

### Immunofluorescence Analysis

Ten thousand PBMECs per well (2 × 10^4^ cells/well) were seeded in 12-well plates. The cells were incubated overnight and then treated according to the experimental design. For the immunofluorescence procedure, PBS (200 μL/well) was used to wash the coverslips covered by cells three times, and then 4% paraformaldehyde (500 μL/well) was added to fix the structure of cells for 15 min. Cells were washed three times with PBS and then perforated with 0.3% Triton X-100 (T9284; Sigma–Aldrich) for 15 min at room temperature to increase the permeability. After washing with PBS three times, the surface of cells was blocked for 1 h with 1 × PBS/5% BSA/0.3% Triton-100 blocking buffer at room temperature. The resultant cells were incubated with the anti-p65, anti-COX2, and anti–HO-1 primary antibodies (same as those used in the Western blot analysis) in antibody buffer (1 × PBS/1% BSA/0.3% Triton X-100) at 4°C overnight, followed by washing three times with PBS, and then were stained with the FITC-conjugated goat anti-rabbit antibody (A0562; Beyotime) for 1 h in the dark at 37°C. The cells were rinsed three times with PBS gently. The nucleus of the cell was stained by DAPI (1 μg/mL) (D8417; Sigma–Aldrich) for 5 min and then washed three times with PBS. The expression of p65, COX2, and HO-1 was visualized using a DMi8 Microsystems GmbH (Leica, Wetzlar, Germany).

### Statistical Analysis

Data were reported as the means ± standard error of the means. Statistical differences between groups and treatments were determined by one-way analysis of variance with Duncan multiple-range tests by IBM SPSS 20.0 Statistics for Windows (IBM Inc., New York, NY, USA). *P* < 0.05 was considered as statistically significant. Study experiments were performed in triplicate, with three replicates in each experiment.

## Results

### Effect of MET on the PBMEC Apoptosis and Met Dose Dependence by Flow Cytometry Analysis

To optimize the Met doses, the flow cytometry analyses of PBMECs pretreated with graded concentrations of Met (1, 2, 3, 5, and 10 mM) for 12 h were used to investigate the apoptosis rate by using an annexin V–FITC assay. As shown in Figure 1A, the apoptotic death rate of PBMECs pretreated with Met doses (1, 2, and 3 mM) did not differ from control (*P* > 0.05), whereas compared with the respective control, Met at 5 and 10 mM have induced PBMECs' apoptotic death rate (*P* < 0.01). Concerning early apoptotic death rates of PBMECs, the proportion of living cells pretreated with 3 mM Met showed statistically similar protective effects to control cells (Figure 1A, *P* > 0.05). Therefore, in the subsequent experiments, the dose of Met 3 mM was chosen as a suitable treatment dose.

### Optimization of Met Concentration by Western Blot Analysis

To confirm the proper dose of Met in this study, we used Western blot analysis to determine the optimum concentration of Met among (0, 1, 3, 5, 10, and 15 mM) Met doses (Figure 1B). Based on COX2 protein expression analysis, 3 mM of Met showed a dramatic decrease compared to the control group (*P* < 0.05).

### Optimizing LTA Concentration by Using qRT-PCR and Western Blot Analysis in PBMECs

qRT-PCR and Western blot analysis were used to measure LTA dose dependence (Figure 2). We measured mRNA expressions of superoxide dismutase-1 (*SOD1*), glutathione peroxidase 1 (*Gpx1*), and *COX2* in PBMECs induced by LTA at different doses (0, 50, and 100 μg/mL) for 6 h, as shown in Figures 2A–C. Moreover, COX2 protein level was measured by using Western blot analysis for PBMECs induced by LTA with various concentrations (0, 10, 20, 30, 40, 100, and 100 μg/mL) for 6 h. Our result showed that PBMECs stimulated with LTA at doses of 0, 50, and 100 μg/mL for 6 h; the *SOD1* expression level revealed no significant difference between groups (*P* > 0.05) (Figure 2A). The Gpx1 protein level had no significant difference at 50 μg/mL of LTA compared to the control group (*P* < 0.05) (Figure 2B). Our results indicated that mRNA levels of *COX2* increased significantly in LTA treatments at both 50 and 100 μg/mL compared to the control group (*P* < 0.05) (Figure 2C). Furthermore, to confirm this, we tested *COX2* protein expression by using Western blot; we observed that the *COX2* level was increased significantly with LTA treatment at 100 μg/mL (Figure 2D). Therefore, from these results, we show that LTA at a dose of 100 μg/mL was a suitable concentration to be used for the following experiments.

### Met Induced Activation of AMPK Signaling Pathway Examined in LTA-Induced PBMECs

**Figure 3:**
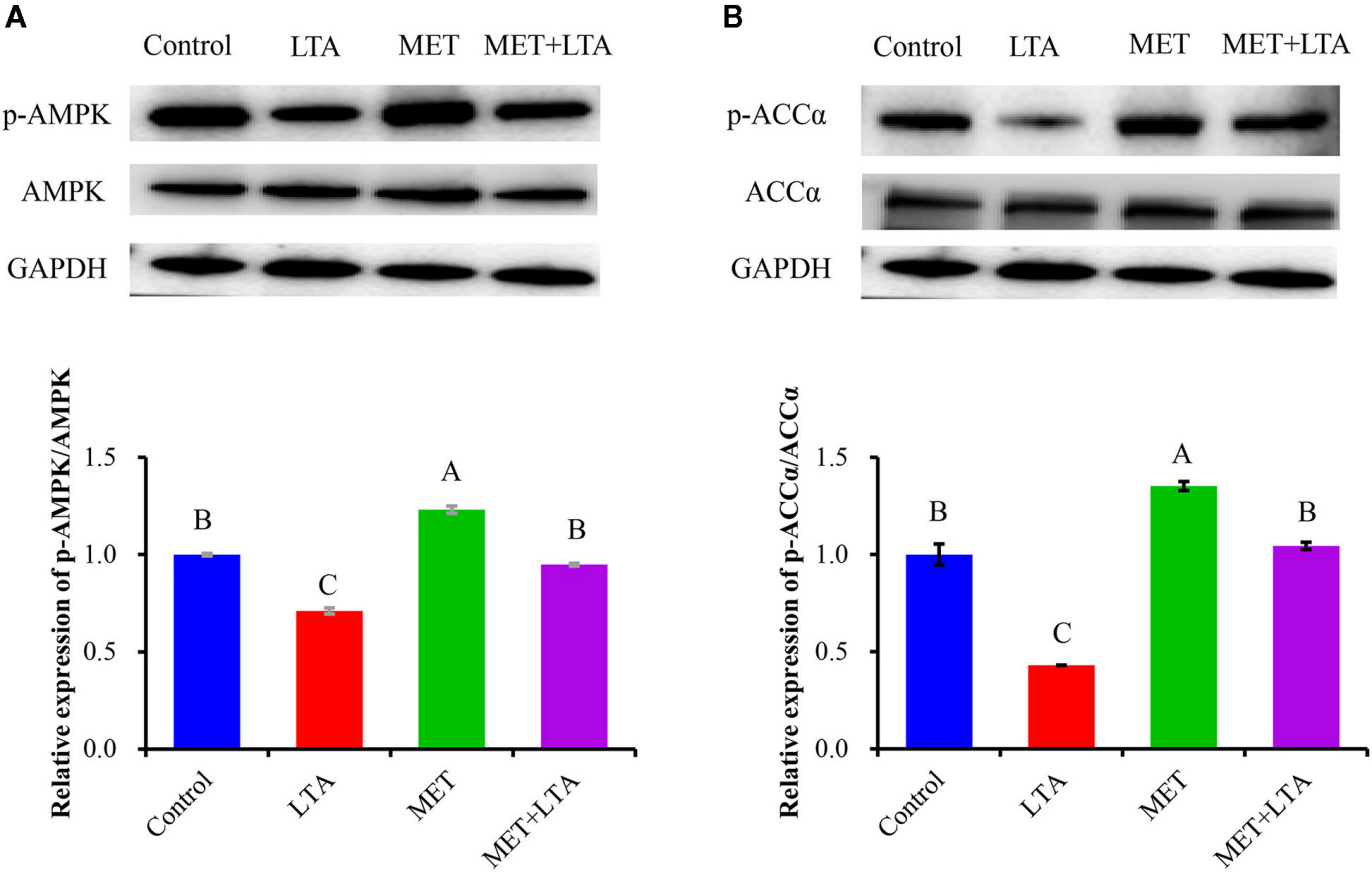
Expression of proteins related to the AMPK signaling pathway. The expression of AMPK signaling–related proteins was measured by Western blot analysis in PBMECs treated with metformin 3 mM for 12 h, and LTA (100 μg/mL) for 6 h. Graphs show densitometric analysis (expressed as P-AMPK ratio to AMPK and P-ACC to ACC) from each band from three independent experiments. Values represent means ± SEM. LTA, lipoteichoic acid; MET, metformin. Different uppercase letters (A–C) on the top of bars indicate significant differences (*n* = 3, *p* < 0.05) vs. among the groups. This study measured the phosphorylation of both **(A)** AMPK and **(B)** ACC expression level.

The protein expressions related to AMPK signaling is shown in Figure 3. The protein levels of phosphorylated AMPKα (P-AMPKα) and ACCα were examined using Western blot analysis. Compared with the control group, the ratio of P-AMPK to total AMPK and the ratio of phosphorylated ACCα to total ACCα (*P* < 0.05) were increased following Met pretreatment (*P* < 0.05). LTA stimulation downregulated phosphorylation of both AMPKα and ACCα compared with the control group (*P* < 0.05). However, the phosphorylated AMPKα and ACCα proteins were increased following pretreatment with Met, which indicated that Met reversed the AMPK signaling inactivation due to LTA treatment (*P* < 0.05). The expression of phosphorylated AMPK was also significantly increased in cells pretreated with Met followed by LTA treatment as compared with the LTA group (*P* < 0.05).

### Pretreatment With Met Suppressed the Inflammatory Response of PBMECs Caused by LTA

**Figure 4:**
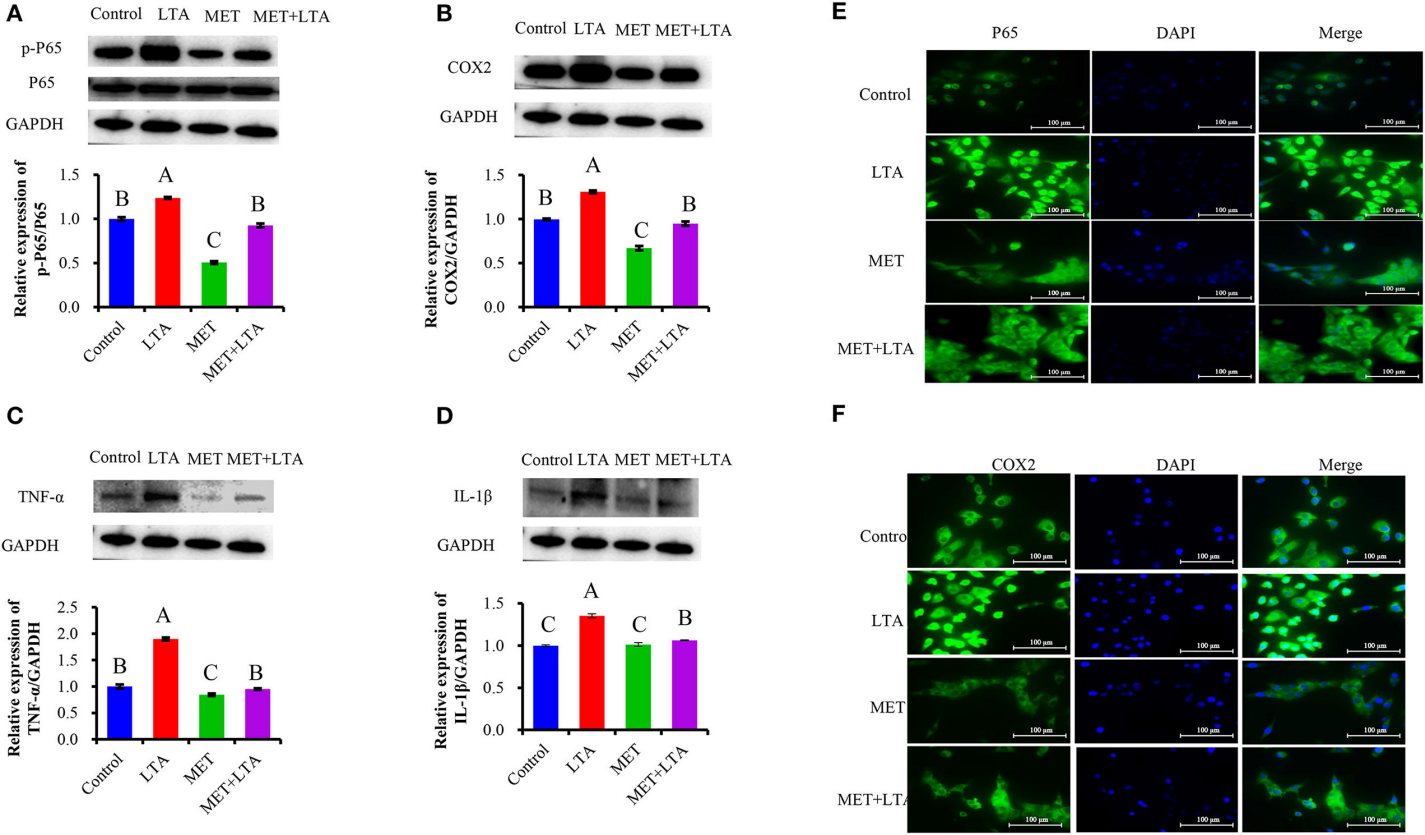
Anti-inflammatory effect of metformin pretreated cells followed by LTA challenge. The expression of NF-κB–signaling related proteins was measured by Western blot analysis in bovine PBMECs pretreated with 3 mM metformin for 12 h and challenged with 100 μL/mL LTA for 6 h. The study measured NF-κB p65 **(A)**, COX2 **(B)**, IL-1β **(C)**, and TNF-α **(D)**, the expression level in the whole-cell lysates. GAPDH was used to normalize the protein expression. Immunofluorescence analysis for NF-κB p65 and COX2 (FITC) was performed, and the nuclear was stained with dye DAPI (blue). **(E, F)** Immunoblots and acquisition of intensity from the respective blots. Western blots shown are the representative image of three independent experiments. LTA, lipoteichoic acid; MET, metformin. Different uppercase letters (A–C) on the top of bars indicate significant differences (*n* = 3, *p* < 0.05) among the groups.

We pretreated the PBMECs with 3 mM Met for 12 h and then stimulated with LTA (100 μg/mL) for 6 h. The protein expressions related to the inflammatory response were measured (Figure 4). Interestingly, we found that the cellular levels of p-p65, IL-1β, TNF-α, and COX2 increased in PBMECs challenged with LTA compared with the control group (*P* < 0.05). However, Met suppressed the expression of phosphorylated p65 (Figure 4A) and downregulated the expression of COX2 (Figure 4B), IL-1β (Figure 4C), and TNF-α (Figure 4D) induced by LTA PBMECs. As seen in Figure 4, compared with the control group, the expression of phospho-p65 in LTA group was prominently increased (*P* < 0.05). However, PBMECs pretreated with 3 mM Met alone or Met combined with the LTA had remarkably lower phospho-p65 than that in control group (Figure 4A). Consistently, the expression levels of COX2, IL-1β, and TNF-α were upregulated in response to LTA stimulation (*P* < 0.05). However, pretreatment with 3 mM Met reversed the LTA induction of cytokines (IL-1β, TNF-α) and COX2 expression (Figures 4B–D).

This study also showed the translocation of NF-κB p65 and subsequently the induction of COX2 in nuclei after LTA induced inflammatory response in PBMECs or pretreatment with Met to reverse the process. Immunofluorescence microscopy was used to visualize the fluorescence signal. FITC fluorescence was used to visualize NF-κB and COX2 (green), and DAPI was used to visualize the nuclei (blue). As our data showed (Figure 4E), compared with control cells, the strongest signal was NF-κB p65, and the green fluorescence was predominantly located in the nuclei in PBMECs stimulated with LTA treatment (Figure 4). However, for cells pretreated with 3 mM Met, there was less NF-κB p65 signal, and it was mainly in the cytoplasm, which was similar to the control treatment. These results indicated that Met played an anti-inflammatory role by preventing the transformation of p65 into the active form, phospho-p65, and translocation into the nucleus. As a result, COX2, the downstream target gene of NF-κB p65 after translocation into the nucleus resulted in a quantitative increase of COX2 expression (Figure 4F).

### Met Regulates NRF2 Pathway and Its Downstream-Regulated HO-1 and Gpx1 Proteins Examined in LTA-Challenge PBMECs

**Figure 5:**
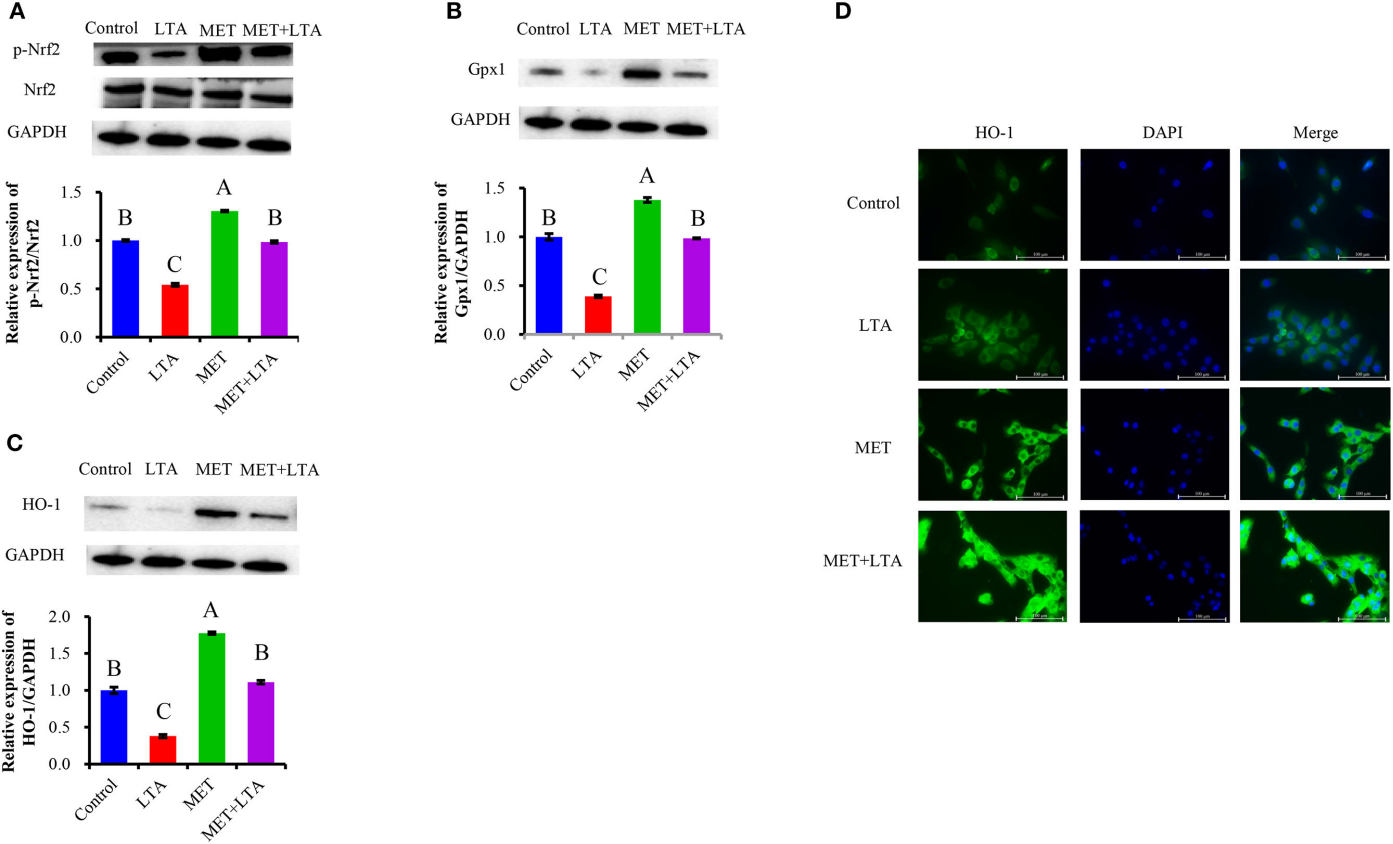
Antioxidative effect of metformin in cells induced by LTA challenge. The expression of Nrf2 signaling–related proteins were measured by Western blot analysis in PBMECs pretreated with 3 mM metformin for 12 h and challenged with 100 μL/mL LTA for 6 h. The study measured the NRF2 **(A)** and its downstream Gpx1 **(B)** and HO-1 **(C)** protein expression. GAPDH was used to normalize the protein expression. Immunofluorescence analysis for HO-1 (FITC) was performed to visualize HO-1 (green), and the nuclear was stained with dye DAPI (blue). **(D)** Immunoblots and acquisition of intensity from the respective blots. Western blots shown are the representative image of three independent experiments. All results are expressed as the means ± SEM. LTA, lipoteichoic acid; MET, metformin. Different uppercase (A–C) on the top of bars indicate significant differences (*n* = 3, *p* < 0.05) vs. among the groups.

We examined Nrf2 and phosphorylated Nrf2 (p-Nrf2) and its downstream HO-1 and Gpx1 protein expression in terms of genes and their coding proteins related to oxidative stress to confirm the antioxidant effects of Met. Western blot analysis demonstrated that Nrf2 protein level was decreased in the PBMECs stimulated with LTA; however, pretreatment with Met upregulated the level of Nrf2 that was downregulated by LTA treatment in PBMECs (Figure 5A). Moreover, the expression of Gpx1 (Figure 5B) and HO-1 (Figure 5C) activation, which decreased in response to LTA stimulation, was significantly upregulated by pretreatment with Met (*P* < 0.05).

To confirm the results from Western blots analysis, the current study determined the downstream protein expression (HO-1) of Nrf2 in the mammary cell using immunofluorescence. FITC fluorescence was used to visualize HO-1 (green), and DAPI was used to visualize the nuclei (blue). As our results showed (Figure 5D), because of location change of upstream kinase of HO-1 after LTA induced oxidative response or pretreatment Met in PBMECs. The weakest signal was HO-1, and the green fluorescence was predominantly located in the cytoplasm in the LTA treatment. For cells pretreated with 3 mM Met, there was the strongest HO-1 signal, and it was mainly in the nuclei, which was similar to the control group.

## Discussion

**Figure 6:**
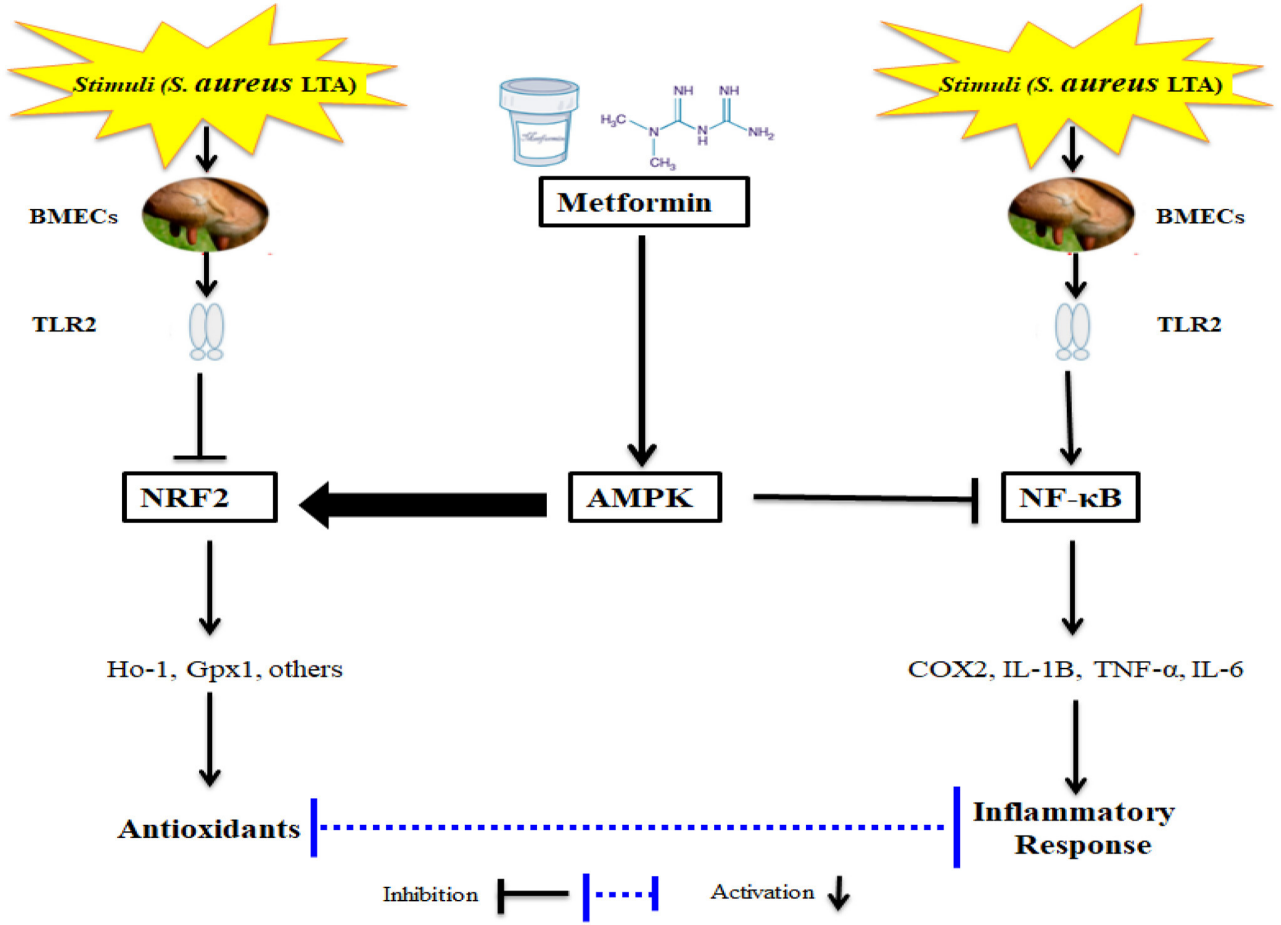
Schematic Representations of Metformin and LTA.

The key motivation of this study was to determine if activation of AMPK via Met has a potential effect in suppressing oxidative stress and inflammatory responses in an LTA-induced mastitis model using isolated BMECs (Figure 6). It is not surprising that Met has been demonstrated to induce an antioxidant response; however, the effect of Met on inflammation and cellular antioxidant protein expression induced by LTA in the PBMECs has not been characterized. Therefore, it is necessary to determine the functional and novel role of AMPK together with the application of Met, which would provide novel strategies besides antibiotics for veterinarians to intervene during cases of acute mastitis. The current study demonstrated a protective effect of Met on LTA-induced inflammation in mammary cells through specific activation of AMPK signaling and inhibition of NF-κB signaling. In addition to the anti-inflammatory role, Met might be involved in the activation of Nrf2 pathway; this activation is markedly AMPK-dependent in LTA-stimulated oxidative stress in PBMECs *in vitro*. Uncovering the functional relationship between AMPK and Nrf2 pathways is of significance because it reveals a novel link between energy homeostasis and inflammation suppression. This finding may substantially contribute to the development of new therapeutic approaches for inflammatory diseases such as mastitis.

LTA, an endotoxin of bacteria embedded in the cytoderm of *S. aureus*, is released during the cell proliferation process and/or after death (12) and was identified to activate inflammatory responses (31). Interestingly, *S. aureus* and its endotoxins' internalization into PBMECs were critical factors associated with the active state of NF-κB and induction of inflammatory responses and affected lactation in the mammary gland of cows (7, 8, 32). Research has demonstrated that LTA inhibited cell proliferation in PBMECs.

As a sensor of intracellular energy status, AMPK is an attractive target for inflammation control. Indeed, there is emerging evidence showing that AMPK activation can decrease oxidative stress and inhibit inflammation (12); another researcher reported that activation of AMPK decreases the production of ROS (33). However, mechanistic connections between AMPK and inflammation have been limited to a link with the NF-κB pathway (13). Several lines of evidence have shown that chemical activators of AMPK decreased NF-κB–mediated transcription in endothelial cells (12). Met exerts its effect by targeting multiple pathways such as activating AMPK and inhibiting mTOR, HER2, and NF-κB pathways (21). Met can induce AMPK phosphorylation, protein kinase c, and mitogen-activated protein kinase directly (34). In this study, LTA stimulation downregulated phosphorylation of both AMPKα and its downstream gene ACCα in PBMECs. However, the levels of phosphorylated AMPKα and ACCα proteins were increased following pretreatment with Met, which indicated that Met reversed the inactivation of AMPK signaling as a result of LTA treatment. The phosphorylation of AMPK expression was also significantly increased under the combined administration of Met with LTA compared to the LTA treatment. Collectively, Met addition activated AMPK signaling via the regulation of phosphorylated AMPK levels. Results of our study agreed with previous research that indicated Met could induce AMPK phosphorylation (34). Other studies showed the dual role of AMPK activation using its inducers such as Met on TNF-α levels in different tissues (22). In mouse neurons, it was indicated that induction of AMPK by its inducers leads to inhibition of COX2, inducible nitric oxide synthase expression and decreased TNF-α, IL-6, and IL-1β through suppression of NF-κB p65 nuclear translocation (23).

NF-κB transcription factor is a key regulator of inflammation and immune responses (35). Met decreased NF-κB activity and reduced proinflammatory cytokine secretion (21), for instance; pretreatment of rats with Met attenuated cellular levels of NF-κB, COX2, and TNF-α, which are considered essential proteins involved in the inflammation pathway (24). Those findings were in part replicated in the current study because Met suppressed the expression of P-p65 in the PBMECs challenged with LTA. The NF-κB downstream protein expression of COX2, IL-1β, and TNF-α upregulated in response to LTA stimulation; however, pretreatment with 3 mM Met reduced IL-1β, TNF-α, and COX2 expression. The results of this study may indicate the protective role of Met in regulating the NF-κB pathway as a potential therapeutic way to treat bovine inflammation.

Another point to be discussed is that it is necessary to expand the investigation on signaling networks other than the NF-κB pathway for inflammation suppression, together with the unequivocal action of Nrf2-mediated antioxidant pathway on ROS clearance. Thus, searching the central probability of a new pathway for inflammation suppression, the downstream pattern of the AMPK pathway might be considered a molecular mechanism. Given this situation and the promising importance of the relationship between energy and redox equilibrium, we planned this line of study by asking specific questions: Is there is potential and functional crosstalk between NRF2 and AMPK pathways? If such crosstalk exists, how do they interact together in inflammation suppression? Studies have previously reported that activation of Nrf2 or AMPK signaling elicit patently overlapping phenotypic cellular or organismal responses and are alongside activated by several natural molecules, indicating cooperation (36, 37). Accordingly, an AMPK-driven boost of the Nrf2 signaling axis has already been confirmed in several studies (38, 39). Only one study has demonstrated direct phosphorylation of Nrf2 by AMPK in an *in vitro* enzyme assay at position Ser 558 (human Nrf2) (40). AMPK phosphorylates Nrf2 at the Ser 550 residue, which, in conjunction with AMPK mediated GSK3 inhibition, promotes nuclear accumulation of Nrf2 for ARE-driven gene transactivation (40). Serving as a stress sensor, AMPK exerts a beneficial effect in the prevention of ROS accumulation to alleviate oxidative stress (15, 16). On sensing redox system imbalance, notably, the AMPK pathway shares distinct association with an antioxidant response, Nrf2, a master regulator that has been activated to upregulate antioxidant gene expression (38). The underlying mechanism is demonstrated to be the induction of AMPK on Nrf2 and its downstream target HO-1 (41, 42). A study using mouse embryonic fibroblasts has shown the AMPK-triggered direct phosphorylation effect on Nrf20 (17). Therefore, to investigate the potential for a functional interaction, we examined the effect of Met on oxidative and inflammatory stress by utilizing LTA-stimulated PBMECs in the present study. Lines of evidence have shown that activation of AMPK by Met stabilized Nrf2 levels, and this phenomenon leads to protection. Therefore, pretreatment with Met increased Nrf2 expression sufficiently to induce ARE genes, which subsequently activated antioxidant related factors such as HO-1, glutathione, and catalase (25). In our work, Nrf2 at the protein level was decreased in the PBMECs challenged with LTA. However, pretreatment with Met upregulated the level of Nrf2 and HO-1 that was downregulated by LTA treatment in PBMECs. Importantly, pretreatment with Met also reversed the translocation of Nrf2 that induced by LTA. To our knowledge, the results of this study indicated the effect of Met on Nrf2 protein in PBMECs induced by LTA, and it may provide an opportunity for a new mechanism on functional molecules capable of interfering with the binding and activation of the Nrf2 pathway.

## Conclusion

In the LTA dose-dependent experiment, the gene expression level of COX2, IL-1β, and IL-6 increased significantly in LTA-challenged PBMECs relative to those in control cells. The protein level of NrF2 was decreased in the cells treated with LTA. Met addition activated AMPK signaling via the regulation of phosphorylated AMPK level. Additionally, in the PBMECs challenged with LTA, the pretreatment with Met attenuated cellular levels of NF-κB, TNF-α, COX2, and IL-1β. Moreover, pretreatment of PBMECs with Met upregulated Nrf2 and HO-1 protein expressions, which were downregulated by LTA treatment. Importantly, pretreatment with Met also reversed the translocation of Nrf2 induced by LTA. Altogether, our results indicate that Met exerts the anti-inflammation and antioxidant effects through regulation of AMPK/NrF2/NF-κB signaling pathway (Figure 6), which highlights the role of AMPK as a potentially therapeutic target for the treatment of *S. aureus*–induced bovine mastitis.

## Data Availability Statement

The raw data supporting the conclusions of this article will be made available by the authors, without undue reservation.

## Author Contributions

AA and TX conceived and designed the experiments. AA and XL performed the experiments. AA and IA analyzed the data. AA, ZC, and AI contributed in figure format. AA, ML, and TX were major contributor in writing the manuscript. ZY support to the financial cost throughout the experiment and generate the ideas. All authors read and approved the final manuscript.

## Conflict of Interest

The authors declare that the research was conducted in the absence of any commercial or financial relationships that could be construed as a potential conflict of interest.
